# Bioarchaeological Insights into the Process of Domestication of Grapevine (*Vitis vinifera* L.) during Roman Times in Southern France

**DOI:** 10.1371/journal.pone.0063195

**Published:** 2013-05-15

**Authors:** Laurent Bouby, Isabel Figueiral, Anne Bouchette, Nuria Rovira, Sarah Ivorra, Thierry Lacombe, Thierry Pastor, Sandrine Picq, Philippe Marinval, Jean-Frédéric Terral

**Affiliations:** 1 CNRS, Centre de Bio-Archéologie et d’Ecologie (CBAE), UMR 5059, Montpellier, France; 2 INRAP Méditerranée, Centre de Bio-Archéologie et d’Ecologie (CBAE), UMR 5059, Montpellier, France; 3 INRAP Méditerranée, Archéologie des Sociétés Méditerranéennes (ASM), UMR 5140, Lattes, France; 4 Université Montpellier 3, Archéologie des Sociétés Méditerranéennes (ASM), UMR 5140, Lattes, France; 5 INRA, Equipe Diversité et Adaptation de la Vigne et des Espèces Méditerranéennes, UMR AGAP, Montpellier SupAgro, Montpellier, France; 6 Université Montpellier 2, Centre de Bio-Archéologie et d’Ecologie (CBAE), UMR 5059, Montpellier, France; 7 CNRS, Archéologie des Sociétés Méditerranéennes (ASM), UMR 5140, Lattes, France; KULeuven, Belgium

## Abstract

Grapevine (*Vitis vinifera*), one of the most important fruit species in the Classical Mediterranean world, is thought to have been domesticated first in South-Western Asia, during the Neolithic. However, the domestication process remains largely unknown. Crucial unanswered questions concern the duration of the process (rapid or slow?) and the related geographical area (single or multiple-origins?). Seeds from domesticated grapevine and from its wild ancestor are reported to differ according to shape. Our work aims, first, to confirm this difference and secondly to identify the extent of domestication in the grapes cultivated by Romans in Southern France during the period 50 BCE–500 CE. We had the opportunity to analyze uncharred waterlogged grape pips from 17 archaeological sites. Based on an extended reference sample of modern wild grapevines and cultivars our work shows that both subspecies can be discriminated using simple measurements. The elongation gradient of the pip’s body and stalk may be regarded as an indicator of the strength of the selection pressures undergone by domesticated grapes. Grapevines cultivated during the Roman period included a mix of morphotypes comprising wild, intermediate and moderately selected domesticated forms. Our data point to a relative shift towards more selected types during the Roman period. Domestication of the grapevine appears to have been a slow process. This could result from the recurrent incorporation into cultivation of plants originating from sexual reproduction, when grape cultivation essentially relies on vegetative propagation.

## Introduction

### Grapevine Domestication and Seed Morphology

Grapevine (*Vitis vinifera* L.) is the most important economic fruit species in the modern world [1]. In 2010, the total harvested area of grapes in the world was estimated, by FAOSTAT, at about 7.1 million ha (http://faostat.fao.org/default.aspx#ancor). During Antiquity, it was already a major fruit crop in the Mediterranean area. Grapevines were cultivated to produce fruits, to be eaten fresh or dried, and especially wine, a drink of great economic, cultural and symbolic value [2], [3], [4], [5]. Today, only a few cultivars are of economical importance but thousands have been described and classified according to their main use: wine, table grapes and dried raisins [6], [7],[8]. How and when has such diversity emerged? Classical authors such as Theophrastus (*Historia Plantarum*, 2), Virgil, (Georgics 2), Columella (*De Re Rustica*, 3) and Pliny the Elder (Natural History, 14) mention the existence of numerous varieties of cultivated grapes. But these types cannot be linked to modern cultivars [9].

The wild ancestor of the domesticated grapevine (*V. vinifera* subsp. *vinifera,* hereafter *V. vinifera*) is well known. *V. vinifera* subsp. *sylvestris,* hereafter *V. sylvestris*, is a heliophilous trailing plant, which thrives in alluvial and colluvial woodlands, from the Himalayas to the Atlantic coast, between the 43^th^ and 49^th^ parallels, North [10], [11], [12], [13].

However, two major and interconnected questions regarding the domestication process and the diversification of grapevines are still debated. Was domestication a rapid process, based on selection of mutants and subsequent propagation by vegetative multiplication or was it a slow process, involving sexual crosses and progressive natural and human selection [14]? Did domestication only occur in a restricted area or did it have a multiple-origin, involving several populations over the distribution range of the wild progenitor [15]?

It is usually assumed that the grapevine was first domesticated in the Caucasus, an area where high morphological diversity is encountered among wild forms and local cultivars [16], [17], [18]. Its cultivation is generally considered as being spread later over the Mediterranean basin due to Bronze Age and Classical societies [5]. SNP genetic data is consistent with a South-West Asian origin of the domesticated grapevine but also suggest introgression of modern Western European cultivars from local wild plants [19]. Other genetic markers even suggest that domestication centers are also to be seen in Italy [20] and in the Western Mediterranean area [15].

It seems important to exploit the potential of archaeology to investigate the origins and the chronology of grapevine domestication, despite the fact that information is still fragmentary and could be considered insufficient to deal with this issue.

Archaebotany provides evidence of the gathering of berries all over the natural distribution area of the wild grapevine, well before the Neolithic and the beginning of agriculture, occasionally even in Pleistocene deposits [21], [22], [23], [24] [25]. The pips being generally mixed with other food waste, it seems probable that the berries were eaten by men, even if other uses cannot be ruled out. The most ancient indicators of wine making activities date back to the Neolithic in South-West Asia. This is the case of the chemical analyses of dried residues found in ceramic vessels regarded as evidence of wine making at Shulaveri-Gora (Georgia), during the 6^th^ millennium BCE [5], at Hajji Firuz Tepe, in the Northern Zagros mountains of Iran, ca 5500-5000 BCE [26] and at Areni Cave, in South-Eastern Armenia, ca 4000 BCE [27]. Remarkably, clear archaeobotanical evidence for the extraction of grape juice is also available in Dikili Tash, Northern Greece, ca 4450-4000 BCE [28]. However, as all these sites are located within the modern distribution range of the wild grapevine, this evidence cannot be considered as proof of domestication or even cultivation. In South-West Asia, no indication of cultivation is available before the 4^th^ - 3^rd^ millennia BCE; thereafter, carbonized pips, berries, pollen and wood remains are more frequently reported from sites located well outside the distribution area of *V. sylvestris* [11], [12], [29]. However, this evidence may represent already a well advanced step in grapevine cultivation and domestication. It is known from other archaeological and written sources that the Bronze Age urban, highly stratified societies emerging at the time, in the area, practiced speculative cultivation of both grapevines and olive trees for wine and oil production [30], [31], [32]. As a matter of fact, domestication of the grapevine in itself is very hard to document from archaeology because of the difficulty in discriminating remains from wild and domesticated grapevines. Both subspecies mainly differ according to their reproductive biology, wild grapevines being dioecious and cross-pollinated while most of the cultivars are hermaphrodite and capable of self pollination [14]. Domestication also induced an increase in the size of both bunch and berries and in their sugar content, thus ensuring greater yields, more regular production and better fermentation [10], [33], [11], [14]. Unfortunately these features hardly leave any archaeological trace. Only seed morphology can help us to trace domestication. Wild grapes bear roundish pips with short stalks while seeds in domesticated varieties are more elongated and with longer stalks [10]. Since Stummer’s work [34] this dissimilarity has been widely used in archaeobotany [35], [36], [37] to distinguish both compartments. Pips displaying domesticated morphology have been occasionally reported from several Neolithic and Bronze Age sites in the Near East and Caucasian area [35], [38]. On the other hand, the wild morphotype is often described in recent Bronze Age, Iron Age or even historical sites in the Eastern Mediterranean and in South-Western Asia, at a time when grapevines had already been cultivated from a long time [39], [35], [40], [41]. Such pips are sometimes considered as representatives of primitive cultivars, which could be regarded as evidence of a slow domestication process. But they are also considered as evidence of either the collection of wild fruits or the inadequacy of morphometry to discriminate wild from domesticated grapes. The use of morphological traits of the pip has actually been strongly criticized and deemed unreliable [42], [40], [11] based on:

- the overlapping of seed shapes
- the deformation of archaeological seeds, due to carbonization, which could make the shape of domesticated seeds similar to wild pips.

This problem requires further thorough investigation, especially regarding the reliability of measurements. In fact, morphometric criteria applied up to now have relied on reference models including few cultivars and wild grapevines originating from restricted areas [40], [37]. These models cannot be considered representative of the diversity of grapevines, especially when applied to other regions [43]. Fortunately, a recent morpho-geometrical approach based on a large sample of cultivars and wild individuals has shown that pips from both subspecies can be discriminated accurately [44].

Similar problems have arisen previously, this time concerning the olive tree; the use of adequate modern reference models was crucial to investigate the domestication of this species based on morphometric and quantitative anatomical analyses of archaeobotanical remains [45], [46], [47].

The aim of this article is to draw attention to the results obtained when applying morphometric analyses to archaeological pips (Roman period), thus exemplifying how bioarchaeology can contribute to the investigation of the domestication process of the grapevine, its duration and geography.

To start with, it was necessary to assess the possibility of discriminating pips belonging to modern cultivars and wild grapevines using traditional measurements and a representative reference collection from various areas of Europe and the Mediterranean. Secondly, we wished to compare the Roman grapes to the modern referential, to evaluate how close the ancient cultivated types are to modern cultivars. Long after the first domestication and far away from its origins, it seemed crucial to understand whether the intensive and highly speculative Roman viticulture used domesticated vines comparable to the modern varieties, or if the domestication process was still under-way.

Recent excavations, carried out in Southern France, yielded great numbers of waterlogged uncharred plant remains, including grape pips. This gave us the opportunity to focus our study on samples of waterlogged pips thus avoiding the problem of deformation due to carbonization [42]. The absence of any visible deformation in waterlogged pips makes it possible to compare directly sub-fossil specimens with their modern counterparts.

### The Archaeological Context of Roman Viticulture in Southern France

According to the written sources, grape cultivation in Southern France began with the foundation of the Greek city of *Massalia*, in 600 BCE [48], [49], [50]. This seems to be confirmed by archaeology which testifies to the start of the city’s production of wine amphora during the 6^th^ century BCE. During the Iron Age, indigenous Celtic populations bought large quantities of wine, which played an important part in their social and political life, featuring largely during feasting [51]. Nevertheless, until the Roman colonization, the adoption of viticulture was slow and restricted to coastal locations [52], [25], [48].

A drastic change occurred during the end of the 1^st^ century BCE [53], [49], when grapevine cultivation gained large territories within the *Narbonensis* province. Wine production was largely promoted by the settlement of Roman army veterans seeking lucrative agricultural activities. The number of large and small scale rural establishments, with their typical wine press and cellars, increased during the 1^st^ century CE. Equipments for wine production were also present in towns and villages. The production of amphorae was carried out all over the Mediterranean part of the province during the 1^st^ and 2^nd^ centuries CE.

The spread of viticulture in South-Eastern France is well illustrated by the multitude of grapevine plantation pits and trenches uncovered recently by rescue excavations over large surfaces. The *Narbonensis* wine was largely exported to other regions in Gaul, to Rome and other areas of the Empire. Amphorae remains used to transport this wine are frequently recovered in far away regions such as Eastern Egypt and India [49]. However, by the end of the 2^nd^ - early 3^rd^ century CE, the wine economy underwent a crisis and many small and middle sized production establishments were abandoned. Comparatively, large scale properties seem to have been spared by the crisis [53], [49].

It is clear that archaeology has provided a considerable amount of information concerning production structures and the chronological dynamics of Roman viticulture. However, the characteristics of the grapes cultivated by the Romans remain largely unknown, despite the abundance of well preserved grape pips recovered these last years. Our study aims at filling this gap in our knowledge.

**Figure 1:**
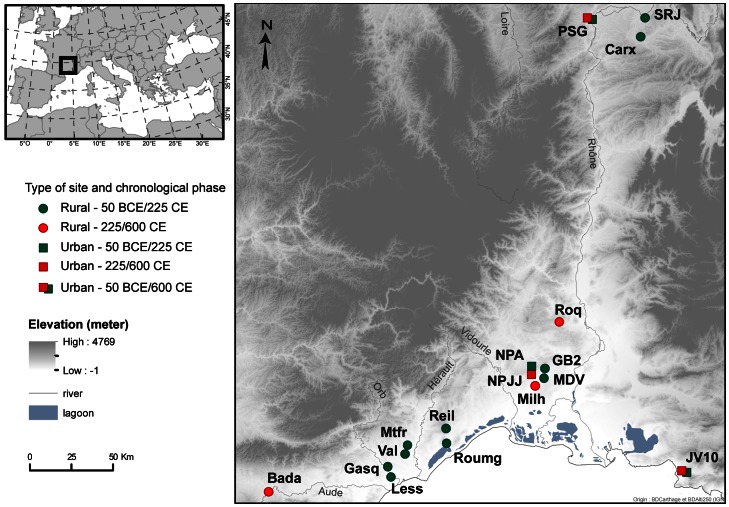
Location of discussed archaeological sites.

The sites taken into account are located either near the Mediterranean coast or in the Rhône valley (Figure 1). The two regions are very different in terms of the bioclimatical conditions, and their viticulture was based on different cultivars during the last centuries. In the South, the climate is hot and dry, typically Mediterranean (average temperature of coldest month above 3°C; average temperature warmest month often above 28°C; average annual precipitation = under 900 mm, with an autumnal peak). In the Rhône valley, mean temperatures are colder especially during the winter (average temperature of coldest month under 0°C in Lyon; average temperature warmest month = 25°C, average annual precipitation = above 800 mm, more regularly distributed). During Antiquity, the two regions also differed concerning the process of wine making. In the South, ceramic vessels were used during fermentation, storage and transport while wooden casks were used in the Rhône valley [49].

## Materials and Methods

Our modern reference material is composed of pips from Euro-Mediterranean traditional cultivars and wild grapevines thriving in their natural habitat and in germplasm repositories. Pips from wild populations have been sampled from 29 female grapevines (*V. sylvestris*) growing in 15 different locations, in different countries (Table S1). Pips from 7 wild individuals preserved in the collections of INRA Colmar (France), INRA Domaine de Vassal, Marseillan (France) and of Rancho de la Merced, Jerez de la Frontera (Spain) were also included. These samples are interesting because, although genetically wild, the plants are cultivated in the same way as the neighboring domesticated grapevines. Therefore they can be considered as cultivated wild grapevines.

We also took into account 84 domesticated varieties originating from a variety of geographic areas in Europe, South-West Asia and Northern Africa (Table S2). The majority of the cultivars are hermaphrodites but some female varieties were also included. The seeds have been collected at the INRA Domaine de Vassal grapevine germplasm collection (http://www1.montpellier.inra.fr/vassal/) and in the field in Turkey. When possible, we analysed 30 seeds per each wild and domesticated individual.

The archaeological pips were recovered from 17 rural and urban Roman and Late-Roman settlements (Figure 1, Table 1). The plant remains were preserved in sediments kept in constant anaerobic conditions, in structures like wells, basins and ditches. Some of the rural sites can clearly be characterized as wine producer settlements, based on the presence of various typical implements (e.g. wine presses, cellars, plantation pits) [53], [49]. Only assemblages of at least 15 pips per stratigraphic unit were taken into consideration. Assemblages are dated based on the associated archaeological artefacts (pottery, coins).

**Table 1:** Origins of waterlogged archaeological assemblages of grape pips.

Code	Site	Type of site	Context	Date	Measurable pips
Carx	Les Cariaux, Frontonas (38)	Rural	Channel, Laisse 2–5	0–225 CE	67
SRJ	Vernai, Saint Romain de Jalionas (38)	Rural	Ditch, T57B	0–225 CE	119
PSG1	Parc Saint Georges, Lyon (69)	Urban	Channel, US1615	75–125 CE	23
PSG2	Parc Saint Georges, Lyon (69)	Urban	Channel, US1356	225–275 CE	49
PSG3	Parc Saint Georges, Lyon (69)	Urban	Channel, US1690	250–300 CE	50
Bada	Lo Badarel, Carcassonne (11)	Rural	Well, PT 2174	400–500 CE	36
Gasq5	Gasquinoy, Béziers (34)	Rural, wine producer site	Well, PT 5027	100–200 CE	49
Gasq3	Gasquinoy, Béziers (34)	Rural, wine producer site	Well, PT 3103	100–200 CE	80
Less1	La Lesse, Sauvian (34)	Rural, wine producer site	Well, PT 3005, US 3063	50-0 BCE	78
Less2a–b	La Lesse, Sauvian (34)	Rural, wine producer site	Well, PT 3009, US 3180–81–83	0–100 CE	44
Val	Rec de Ligno, Valros (34)	Rural	Well	100–200 CE	50
Mtfr	Montferrier, Tourbes (34)	Rural, wine producer site	Well, PT 2052, US 2077	100–200 CE	49
Roumg	Roumèges, Poussan (34)	Rural, wine producer site	Well, PT 5001	25–150 CE	25
Reil	La Reille, Montbazin (34)	Rural, wine producer site	Well, US 6022–23	0–100 CE	39
Milh	Careiron & Pesquier, Milhaud (30)	Rural	Well/Dolium, PT 1087/Dol 1248	375–400 CE	17
NPJJ	Place Jean Jaurès, Nîmes (30)	Urban	Well, PT 10002, US 10198	375–600 CE	50
NPA	Place d'Assas, Nîmes (30)	Urban	Well, PT 3094, US 3149	60–70 CE	50
GB2	Georges Besse 2, Nîmes (30)	Rural	Basin, US 4409	100–200 CE	50
MDV1	Mas de Vignoles XIII, Nîmes (30)	Rural	Well, PT 2077	100–200 CE	49
MDV2	Mas de Vignoles XIII, Nîmes (30)	Rural	Well, PT 2176	100–200 CE	49
Roq	La Roquette, Cavillargues (30)	Rural	Well	275–350 CE	50
JV10A	Place Jules Verne 10, Marseille (13)	Urban	Harbour, US 107	100–200 CE	49
JV10B	Place Jules Verne 10, Marseille (13)	Urban	Harbour, US 85	150–200 CE	75
JV10C	Place Jules Verne 10, Marseille (13)	Urban	Harbour, US 73	300–325 CE	50

**Figure 2:**
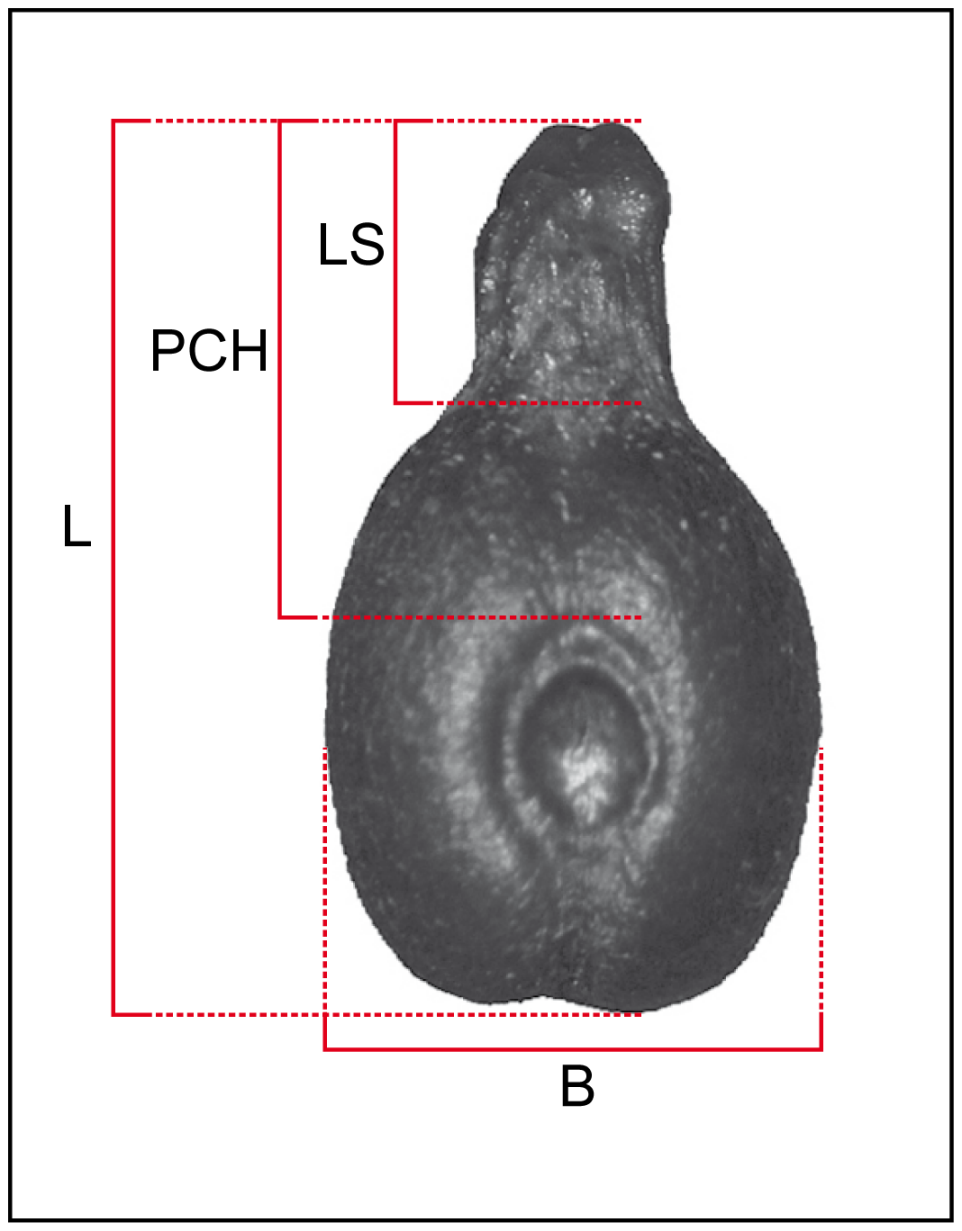
Dorsal view of a pip from domesticated grapevine with indication of morphometric measurements. (L) total length, (LS) length of the stalk, (PCH) placement of chalaza, (B) total breadth.

Modern and archaeological seeds have been photographed in dorsal view using an Olympus SZ-ET stereomicroscope and an Olympus DP 12 camera. Measurements were obtained using Image J v. 1.31 (available as freeware from http://rsbweb.nih.gov.gate1.inist.fr/ij/). According to the literature [37], [36] we selected 4 measurements regarded as the most efficient to discriminate between wild and domesticated grapevines and easily observable on archaeological pips: total length (L), length of stalk (LS), position of the chalaza (PCH) and total width (B) (Figure 2).

In order to minimize the effect of size and to focus on shape information, discrete measurements were converted into log-shape ratios in which each variable is divided by the geometric mean of all 4 variables and then log-transformed [54], [55]. Seed shape variability between and among wild and domesticated grapevines was analysed through a PCA performed on log-shape ratios. A subsequent UPGMA cluster analysis was performed on wild individual and cultivar centroid coordinates on the PCA axes explaining the highest level of morphological variability (PC 1 and 2). All statistical analyses have been performed using XLSTAT 2012 (AddinSoft, Paris). Waterlogged archaeological pips are plotted as additional individuals in the PCA for direct comparison with modern grapevines.

A complementary Linear Discriminant Analysis (LDA) was performed on log-shape ratios to assess the discrimination between wild and domesticated modern pips, and to test the validity of the sub-groups identified by PCA and subsequent Cluster Analysis. Secondarily, archaeological specimens are statistically classified as additional individuals using the LDA discriminating wild and domesticated grapevines.

The relation between pip size, shape parameters and berry size was studied on a sample composed of 72 berries from 3 Italian wild grapevines and 201 berries from 10 different table and wine cultivars. The number of pips per berry was recorded and pips were measured according to the procedure described above. Each berry contained 0 to 4 seeds. When more than 1 pip was present, we considered mean measurements of all pips for each berry. Wild grapevine berries are spherical. In our sample, height and diameter of berries from cultivars are highly correlated (R Pearson = 0.864; p<0.0001; R^2^ = 0.746). Therefore we only considered the diameter as descriptor of berry size.

## Results and Discussion

### Pip Shape in Wild and Domesticated Grapevines

**Figure 3:**
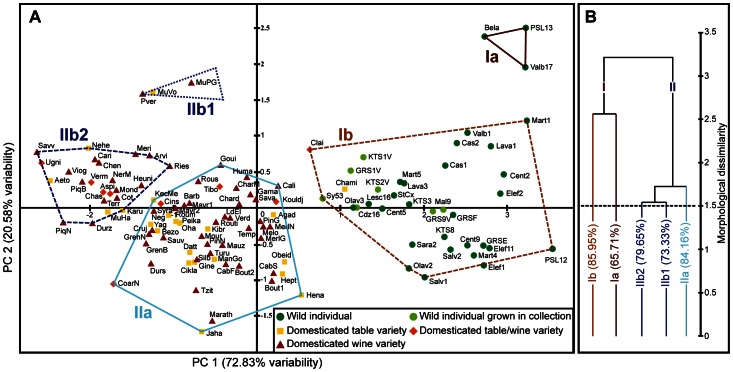
Principal Component Analysis of modern cultivars and wild individuals and subsequent Cluster Analysis (UPGMA) based on centroid coordinates on PC1 and 2. A, PCA biplot of axes 1 and 2 (93.41% variability). For more clarity only the centroid of each group is represented. B, UPGMA dendrogram representing sub-groups identified at an arbitrary Euclidian distance of 1.5 (dotted line) with indication of the discriminant rate (%) calculated by LDA. For composition of sub-groups see A.

Morphometric analyses of the modern reference sample confirm the existence of seed shape differences between *V. vinifera* and *V*. *sylvestris*. The first two principal components (PCs) of the PCA explain 93.4% of total variance (Figure 3, A). PC1 (72.83% of variance) separates cultivars from wild individuals. It is principally correlated to the variables Log-Shape LS, negatively (R = −0.942), and Log-Shape B, positively (R = 0.955). It express the opposition between roundish pips with a short stalk, typical of *V. sylvestris*, and more elongated pips with a long stalk that are clearly connected to the domesticated compartment. PC2 (20.58% of variance) is linked to the position of the chalaza (PCH) (R = 0.824).

The two main clusters in the UPGMA dendrogram (I and II) reflect the major discrimination between the wild and the domesticated grapevines (Figure 3, B). One cluster is composed of cultivars only while all wild grapevines are clustered in the second group which, in addition, includes solely two cultivars (Chami and Clairette). A subsequent LDA confirms the strong discrimination between pips from wild and domesticated grapevines. The model shows that 94.4% of the pips are correctly re-assigned (96.69% for domesticated and 88.62% for wild grapevines).

The sub-groups that can be further identified by the dendrogram cannot be interpreted according to geographic origin of wild and domesticated grapevines, to environmental parameters, to main uses or berry color of cultivars (Figure 3, B, Table S1 and S2). Wild individuals from natural habitats and from collections are not significantly discriminated even if the last ones extend towards domesticated varieties in the PCA biplot. This trend may be linked to environmental factors, cultivation practices ensuring better growing conditions for the plants sampled from living collections. An impact on pip size could be sufficient to explain the small variation between both groups. Actually, the log-shape ratios method allows us to eliminate isometric size but not allometric size [56].

### Pip Shape and Domestication

Pip shape clearly changed with domestication. However, it cannot be assumed that morphological features have been a target of conscious selection. Therefore, changes in shape are more likely connected to selected characters by a pleiotropic effect [44], [57]. Berry size can be expected to be one of these characters as it underwent drastic changes under domestication. The diameter of wild grapevine berries is generally inferior to 0.8 cm while it ranges from less than 0.8 cm to over 3, near 4 cm, in the domesticated compartment [8].

Negrul [58] observed a correlation between berry and pip size. Our results confirm the existence of a relation between the diameter of berries, the number of pips per berry, pip size and shape variables (Table 2). However, and most importantly, our results provide evidence of a contrasted situation between wild and domesticated grapevines. If the correlation between berry diameter and the number of seeds per berry is moderate for the wild grapevine, it appears to be weak for cultivars. A similar situation is observed concerning the correlations between berry diameter and pip size (expressed by maximum length) and shape parameters (Log-Shape LS and Log-Shape B), always stronger for wild grapevines. In the wild compartment, small berries produce typical small and roundish pips while large berries produce larger and more elongated pips, with a longer stalk. Therefore, selection of grapevines producing large berries probably induced elongated pips. Weaker correlations between berry size and pip parameters in the domesticated compartment probably reflect an additional effect of domestication related to the selection of fruits with a higher proportion of flesh regarding the volume of pips.

**Table 2:** Correlation values between berry size and pip number, size and shape parameters.

	Wild Compartment	Domesticated Compartment
Number of pips/Berry diameter	R Spearman = 0.728; **p value <0.0001**; R^2^ = 0.530	R Spearman = 0.171; **p value = 0.015**; R^2^ = 0.029
L/Berry diameter	R Spearman = 0.827; **p value <0.0001**; R^2^ = 0.684	R Spearman = 0.288; **p value <0.0001**; R^2^ = 0.083
Log Shape LS/Berry diameter	R Spearman = 0.510; **p value <0.0001**; R^2^ = 0.260	R Spearman = 0.194; **p value = 0.006**; R^2^ = 0.038
Log Shape B/Berry diameter	R Spearman = −0.725; **p value <0.0001**; R^2^ = 0.526	R Spearman = −0.229; **p value = 0.001**; R^2^ = 0.052

The elongation gradient of pip body and stalk may be considered as a continuous domestication syndrome. Variations amongst cultivars (Figure 3) may illustrate a domestication gradient inside the domesticated compartment. As a matter of fact, ampelographic criteria allow us to recognize, in all traditional European groups of cultivars, primitive varieties resembling wild grapevines (i.e. characterized especially by small bunches and berries and high polymorphism) and more strongly domesticated cultivars [10]. These last ones are characterized by large bunches and berries, high fertility and productivity. They are more sensible to climate, pests and diseases, and display a higher variety both in shape and taste [18], [10]. Statistic multivariate analysis performed on ampelographic parameters confirms that cultivars may be classified according to a gradient that mainly reflects the selective pressures they underwent [59].

### Origins of Archaeological Pips

The assemblages of pips measured here are considered as human refuse; they were found in association with other residues from human waste, in different habitat contexts. Type and quantity of different grape remains may help us identify the precise human activity they derive from. For example, the association of pips, undeveloped berries, fragments of crushed skins, pedicels and other rachis elements is regarded as direct evidence of grape pressing [25], [60], [61].

In our case, the best evidence of such activity is provided by the assemblages found in settlements identified otherwise as wine producer sites (Gasquinoy, La Lesse, Mont Ferrier) (Table 3). In this case, our pips are considered as local production, as it is difficult to envisage that wine could be produced with grapes cultivated far away. Furthermore, plantation pits were present and excavated close to the 3 sites. Other rural sites, such as Lo Badarel, Careiron and Pesquier or la Roquette, delivered assemblages which probably also include wine making residues. Grape pips are strongly represented among other cultivated plant remains. There are significant proportions of pedicels and usually undeveloped grape berries. On the other hand, in urban sites, grape pips generally represent a minor proportion of cultivated plant remains, the number of pedicels in relation to the number of pips is low and other grape remains are rare. As a result, pips are in this case likely to originate primarily from food consumption even if the whole of the refuse remains may include a minor proportion of wine making waste. In this situation, fruits may derive from local production or from trade.

**Table 3:** Abundance of various grape remains in waterlogged archaeological assemblages.

Code	Type	Min totalnb of pips	Nb ofpedicels	Bunchrachis	Grapeskins	Undeveloppedberries	% grape pips/total nb cultivatedplant remains	
Carx	Rural	134	0	–	–	X	90.54	
SRJ	Rural	736	11	–	–	–	37.90	
PSG1	Urban	47	4	–	–	–	1.80	
PSG2	Urban	83	37	–	–	X	3.74	
PSG3	Urban	212	11	–	–	–	14.02	
Bada	Rural	126	22	–	–	–	52.50	
Gasq5	Rural, wine producer site	190	146	–	XX	X	49.35	
Gasq3	Rural, wine producer site	2848	242	XX	XX	X	90.01	
Less1	Rural, wine producer site	461	27	–	X	X	53.17	
Less2a–b	Rural, wine producer site	1846	82	–	XX	–	90.85	
Val	Rural	97	0	–	–	–	92.38	
Mtfr	Rural, wine producer site	1136	143	X	XX	XX	66.43	
Roumg	Rural, wine producer site	NOT AVAILABLE						
Reil	Rural, wine producer site	NOT AVAILABLE						
Milh	Rural	54	6	–	–	X	64.29	
NPJJ	Urban	153	12	–	–	X	12.23	
NPA	Urban	215	10	–	–	–	4.34	
GB2	Rural	189	0	–	–	–	30.63	
MDV1	Rural	948	57	–	–	–	56.03	
MDV2	Rural	235	0				30.17	
Roq	Rural	188	48	–	–	X	48.70	
JV10A	Urban	664	26	–	–	X	4.64	
JV10B	Urban	1215	14	–	–	X	10.46	
JV10C	Urban	150	63	–	–	X	14.72	

### Morphometric Comparisons between Modern and Archaeological Pips

**Figure 4:**
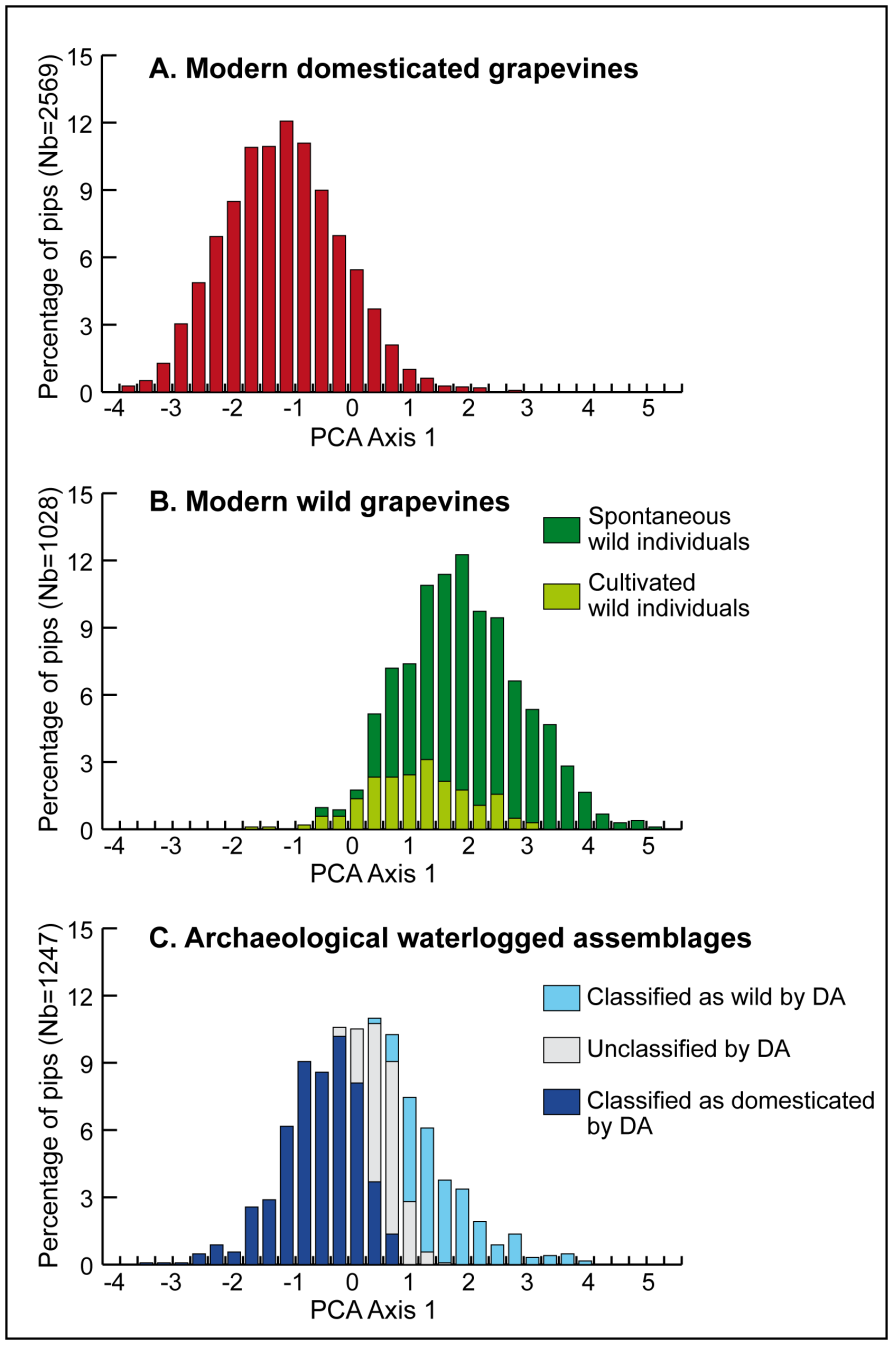
Comparison of modern domesticated and wild grapevines with Roman archaeobotanical assemblages of waterlogged pips. Archaeological pips are plotted as additional individuals in the PCA performed on modern individuals. The distribution of pips on PCA Axis 1 is represented as percentage values according to (A) modern domesticated grapevines (84 cultivars; 2569 pips), (B) modern spontaneous (29 individuals; 818 pips) and cultivated (7 individuals; 210 pips) wild grapevines, (C) archaeological assemblages (17 sites; 1247 pips). Additionally the classification of archaeological pips by DA as wild or domesticated (p value >0.75) is represented (C), see Table 4.

**Figure 5:**
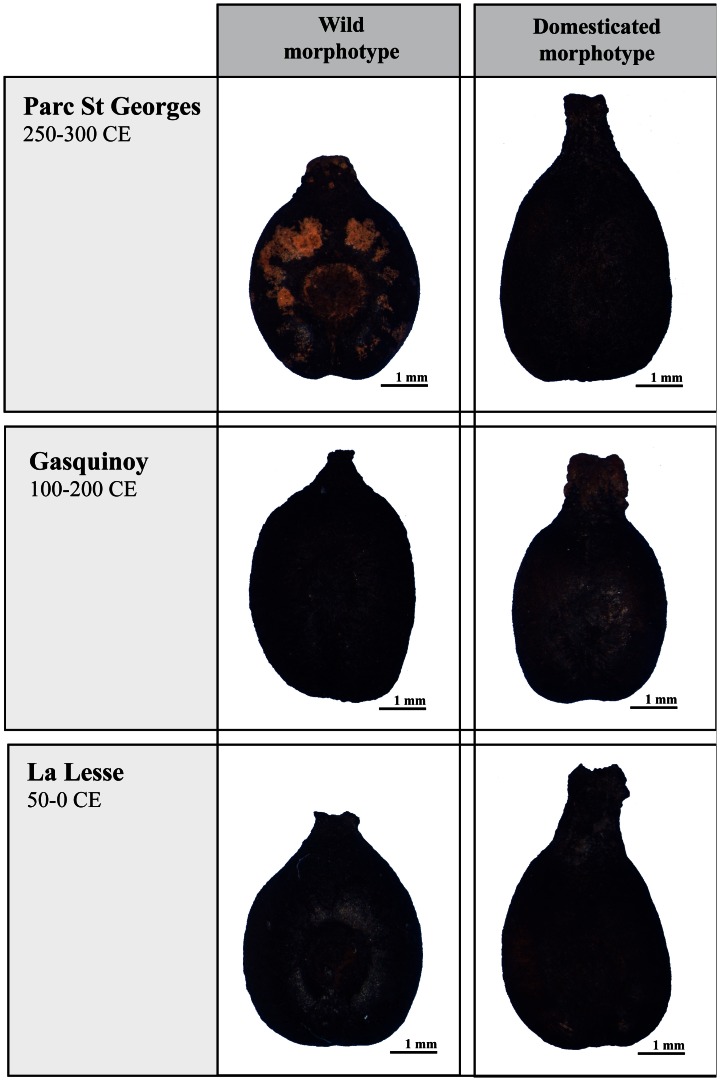
Examples of waterlogged archaeological pips allocated by the LDA to the wild and domesticated morphotypes. Origin: Parc Saint Georges (Lyon, Rhône), Gasquinoy (Béziers, Hérault), La Lesse (Sauvian, Hérault).

Plotted as additional individuals in the PCA, the distribution of the archaeological pips partly overlaps the one from both the domesticated and the wild modern compartments (Figure 4). Archaeological pips don’t cover the whole range of domesticated grape, the varieties with the most elongated stalks, those which are supposed to be the more strongly domesticated, find no subfossil equivalent. Among the values typical of wild grapevines, archaeological pips only recover the range where we can find seeds from modern cultivated wild individuals along with plants growing spontaneously. The higher scores for archaeological pips occur in the intermediate zone between the modern wild and the domesticated compartments. On the other hand, the LDA allocates the majority of the waterlogged pips to the domesticated compartment (54.77%) but a significant proportion (24.22%) is classified as wild (Table 4, Figure 5). Pips unclassified by the LDA are located in the intermediate area between wild and domesticated groups on the PCA biplot (Figure 4), possibly because no modern analogues are found in the reference samples.

**Table 4:** Allocation of archaeological grape pips by the DA to the domesticated and the wild compartments (p>0.75).

Code	Type	Date	Measurablepips	Classified asdomesticated	Classifiedas wild	Unclassified
Carx	Rural	0–225 CE	67	40	16	11
SRJ	Rural	0–225 CE	119	73	25	21
PSG1	Urban	75–125 CE	23	15	5	3
PSG2	Urban	225–275 CE	49	28	7	14
PSG3	Urban	250–300 CE	50	29	8	13
Bada	Rural	400–500 CE	36	26	4	6
Gasq5	Rural, wine producer site	100–200 CE	49	23	18	8
Gasq3	Rural, wine producer site	100–200 CE	80	35	21	24
Less1	Rural, wine producer site	50-0 BCE	78	33	32	13
Less2a–b	Rural, wine producer site	0–100 CE	44	33	5	6
Val	Rural	100–200 CE	50	25	10	15
Mtfr	Rural, wine producer site	100–200 CE	49	26	11	12
Roumg	Rural, wine producer site	25–150 CE	25	9	7	9
Reil	Rural, wine producer site	0–100 CE	39	18	10	11
Milh	Rural	375–400 CE	17	11	5	1
NPJJ	Urban	375–600 CE	50	44	3	3
NPA	Urban	60–70 CE	50	20	21	9
GB2	Rural	100–200 CE	50	31	7	12
MDV1	Rural	100–200 CE	49	10	22	17
MDV2	Rural	100–200 CE	49	19	16	14
Roq	Rural	275–350 CE	50	37	5	8
JV10A	Urban	100–200 CE	49	34	9	6
JV10B	Urban	150–200 CE	75	27	30	18
JV10C	Urban	300–325 CE	50	37	5	8

### Roman Cultivation and the Domestication Process of Grapevine

Part of the archaeological pips classified ‘wild’ by LDA may originate from gathering of berries from wild grapevines growing in the vicinity of the sites. In Southern France, wild grape fruits were already consumed by man at least since the Mesolithic [25]. *Vitis sylvestris* still grows nowadays in Mediterranean France, albeit very sporadically [62], [63]. However, it is thought to have been much more common in the past, before extensive alteration of its habitat by human activities and before the introduction of American pathogens (Mildew, Phylloxera), which devastated European vineyards at the end of the 19^th^ century CE [64], [65]. Although exceptional today, wild grape berries were occasionally collected and even used to make wine, vinegar and to color wine, during recent centuries in the Western Mediterranean [66], [67].

However, archaeological pips allocated to the wild morphotype are too common in our assemblages (30.66% of the total number of classified pips, Table 4); gathering activities could not account for such a considerable and recurrent presence. They are to be found in all the sites, generally in moderate or high proportions (from 6.38 to 68.75% of classified pips, Table 4), and systematically associated with the domesticated type. The wild morphotype is no less represented in urban settlements (Table 5) where it is unlikely that a significant amount of food resources might originate from wild gathering. On the other hand, it is very common in specialized wine producer sites, such as Gasquinoy, where plantation pits cover about 75% of the surrounding landscape, which means that grapes used was certainly grown locally. Wild morphotype pips are therefore, in their majority, an outcome of grape cultivation. The morphological continuity of archaeological pips encompassing part of the wild and domesticated compartments is another indication that the Romans from the *Narbonensis* Province used to cultivate a mix of grapevines, ranging from morphologically wild to domesticated types, and including intermediate forms between modern wild and domesticated subspecies. Grapes cultivated had not yet sustained selective pressures as strong as those underwent by modern varieties. The rarity of elongated pips is evidence of the absence - or of the small importance - of the modern most highly selected grape types.

**Table 5:** Mann-Whitney test results (alpha = 0.05) concerning the proportion of archaeological pips allocated to the wild grapevine by DA according to chronology, geographic situation and type of site.

	U	Espérance	Variance (U)	p-value
Chronology; 50 BCE-225 CE *vs.* 225–600 CE	104.000	59.500	247.809	**0.003**
Geographic situation; Northern Rhône valley *vs.* Mediterranean	37.000	47.500	197.831	0.479
Type of site; Rural *vs.* Urban	80.500	64.000	266.551	0.380

As most of fruit trees, vegetative propagation (rooting, layering, grafting) is favored for grapevines in order to fix desired traits and to reproduce individuals with selected features. Fruit trees are generally highly heterozygous and have a long juvenile phase, which require a long wait (3 to 5 years for grapevine) before fruits can be evaluated. Consequently, selected types cannot be maintained by sexual reproduction. The shift from sexual reproduction in wild populations to clonal propagation under cultivation is regarded as the cornerstone of the domestication syndrome of most fruit trees [68], [12], [69]. In this way, grape cultivation traditionally relies on vegetative propagation while somatic mutations are regarded as having played a crucial role in the emergence of new cultivars [14]. It is therefore surprising to see, from the archaeobotanical evidence, that Roman grape cultivation was not focused on the most selected grape types of the time but included wild types and intermediate forms. We can therefore consider that, not only were the Roman cultivators less selective, but that the diversity of cultivated grape was probably much more dynamic than it is today and that the cultivated compartment was regularly rejuvenated. The emergence of primitive forms could be explained by the regular incorporation into cultivation of a proportion of plants originating from sexual reproduction. In grapevine [10], [11], like in many other fruit trees [68], [70], due to its high level of heterozygosity, progeny originating from seed segregates into a diversity of forms, including some looking like wild forms, even when seed is taken from elite clones. The contribution of sexual reproduction could result from the transplantation of unintentional seedlings bearing desirable traits; noticed by farmers in the wild or near their fields and dwellings. Deliberate sowing could also have been used. In recent times, the transplanting of wild grapevine plants seems rare but it has nevertheless been observed in Central Asia [71]. Latin authors were well aware of the interest of clonal propagation. Pliny the Elder (1^st^ c. CE) strongly advocated against the reproduction of grapes by sowing (*Natural History*, 17, 10). However, Cato the Elder (2^nd^ c. BCE) describes the habit of cultivating specific varieties beside a mix of genotypes with no particular name and referred to under the term *Miscella* (*De Agri Cultura*, 6), which actually could be seedlings. Later on, during the 12^th^ c. CE, the Hispano-Muslim author Ibn al-Awwam advises on the propagation of grapevine by various vegetative methods and also by sowing seeds chosen from selected cultivars (*Kitab al-fila-hah*, 7, 45–46). On the other hand, genetic nuclear SSR markers bring evidence that many modern cultivars have arisen from crosses between cultivated varieties [72], [73], [74], [75], [76].

The regular incorporation of seedlings into cultivation in the past could have favored gene flow between *Vitis sylvestris* and cultivated varieties. The local contribution of wild grapevine in the creation of the domesticated compartment is defended on the basis of chloroplast and nuclear SSR data, in various Mediterranean areas [20], [15], [77].

### Spatio-chronological Dynamics of Grape Diversity

The various morphotypes identified in our study appear to have been jointly cultivated and used for the same purposes during the Roman period. The combination of wild and domesticated morphotypes in wine pressing residues from producer settlements strongly suggests that they were cultivated locally and combined to make wine. Their association in urban rubbish deposits probably implies that they were also eaten as table fruit. The morphometric method used does not detect any difference between pips recovered from urban and rural settlements (Table 5 and 6). Archaeological pips from sites in the Rhône valley differ significantly, on PC 1 and 2, from those originating from the Mediterranean area (Table 6). This is probably due to the bioclimatic differences between both regions. The grape varieties traditionally cultivated in these areas are not identical and still include several typical original cultivars. Morphometrical results may indicate that the adaptation to regional conditions had already begun during Roman times. This would mean that the grapes cultivated in the Rhône area were not simply introduced from the Mediterranean area, where viticulture started earlier, or else that they had already evolved since their introduction.

**Table 6:** Kolmogorov-Smirnov test results (alpha = 0.05) concerning the distribution of archaeological pips on axes 1 and 2 of the PCA according to chronology, geographic situation and type of site.

	PCA Axis 1		PCA Axis 2	
	D	p-value	D	p-value
Chronology; 50 BCE-225 CE *vs.* 225–600 CE	0.203	**<0,0001**	0.050	0.597
Geographic situation; Northern Rhône valley *vs.* Mediterranean	0.121	**0.002**	0.129	**0.001**
Type of site; Rural *vs.* Urban	0.080	0.060	0.056	0.345

However the more remarkable aspect is linked to chronology. It is documented only on the first axis of the PCA (Table 6). Pips dating from the second part of the Roman period and the very early Middle-Ages (225–600 CE) tend to be located towards the negative part of PC 1, where modern cultivars are to be found, while earlier Roman pips (50 BCE-225 CE) are globally shifted towards the wild grape space. For that matter the proportion of pips allocated to the wild grapevine is inferior during later Roman times. Such organization probably underlines the progressive shift towards more highly domesticated cultivated grape forms. The change could have been favored by the crisis which affected the wine economy during the end of the 2^nd^ – early 3^rd^ century CE. The reduction in wine production possibly allowed cultivators to focus on more selected grape types.

## Conclusions

Based on an extended modern reference sample, this study demonstrates that simple measurements of pips can be used to discriminate domesticated and wild subspecies of the grapevine. From wild, ‘primitive cultivars’ to ‘highly domesticated cultivars’, the gradient in the elongation of the body and, more especially, of the stalk of the pip may be considered not only as a domestication syndrome but also as an indicator of the strength of selection pressures. The reason(s) of this change remains unknown. It can only be partly explained by the selection of bigger berries.

When applied to archaeological assemblages of grape pips from 17 sites in Southern France, the morphometrical analysis testifies to the diversity of *Vitis* cultivated during the Roman period, with a mixture of grape types, ranging from morphologically wild to domesticated and including various intermediate forms. During that period, no equivalent to the modern highly domesticated varieties has been identified. Diversity is similar in urban and rural settlements. Evidence from wine producer sites supports the hypothesis that all these grape types were associated to make wine and that they were cultivated locally. Their presence in urban contexts points to food consumption.

Our results provide evidence that, at least some 4 millennia and thousands of kilometers away from primary domestication in South-West Asia, the grape domestication process was still continuing in the Western Mediterranean area. Morphometric evidence shows a relative shift towards more selected types during the very Roman period, possibly in relation with the crisis of the wine economy and subsequent reduction in grape cultivation. The domestication of the grapevine can therefore be seen as a slow and ongoing evolutionary process, which reminds us of the situation recently described for various cereals and pulses in the Near East, East Asia and Western Africa. Due to new information from archaeobotany and genetics, their domestication is now regarded as a complex, long-term and multi-loci process; cultivation apparently started in different locations, first with morphologically wild plants only, before the slow and progressive appearance of domesticates [78], [79], [80], [81].

On the other hand, the hypothesis of a slow domestication process of the grapevine contrasts with the prevailing conception concerning the domestication of fruit trees, which stresses that very few recombination (and selection) cycles could theoretically separate domesticated varieties from their wild ancestors; the domestication of most clonally propagated perennial species is thus considered as a simple, rapid and intentional operation [68], [70]. We hypothesize that the slower than expected rhythm of grapevine domestication is related to the role of sexual reproduction, more important than previously thought. The role of occasional sexual reproduction is documented in detail for cassava (*Manihot esculenta*), a tuberous crop usually propagated by stem cuttings in traditional Amerindian agriculture, and is probably underestimated for other clonally propagated crops [82]. It is difficult to understand why the Romans also cultivated a proportion of weakly domesticated grapevines. One explanation could be related to the reproductive biology of these anciently cultivated grapevines. The majority of the modern varieties produce fruits because they are hermaphroditic and self-compatible, traits of the domestication syndrome [10], [33], [11]. In wild populations, female plants only produce fruits if they are pollinated by male individuals. It is impossible to estimate the proportion of hermaphroditic and self-compatible varieties during Roman times. It is likely that pollination and fruiting were favored by diversity and more especially by the presence of primitive forms in Roman vineyards, which in return could have slowed down the generalization of hermaphroditism.

## Supporting Information

Table S1 Origin of the wild grapevines included in our reference sample. Bioclimatic contexts mentionned are based on [83].
(XLS)

Table S2 Cultivars of our modern reference sample and their main characteristics (Galet 2000, http://bioweb.ensam.inra.fr/collections_vigne/reseau.php?cle=BRG). Use: W = wine; T = table grape. Sex: H = hermaphrodite; F = female; U = Unknown. Geographic area: BA = Balkans; WA = Western Asia; CE = Central Europe; FR = France; IT = Italy; ANE = North Africa/Near East; IB = Spain/Portugal; UN = Unknown. Berry diameter: 1 = from 8 to 12 mm, 2 = 12 to 18 mm, 3 = >18 mm.
(XLS)
